# Supplementary figures and images for: Transient boosting of action potential backpropagation for few-shot temporal pattern learning
Source: PLoS Comput Biol. 2025 Dec 5;21(12):e1013777. doi: 10.1371/journal.pcbi.1013777 (PMC12698000; doi:10.1371/journal.pcbi.1013777)

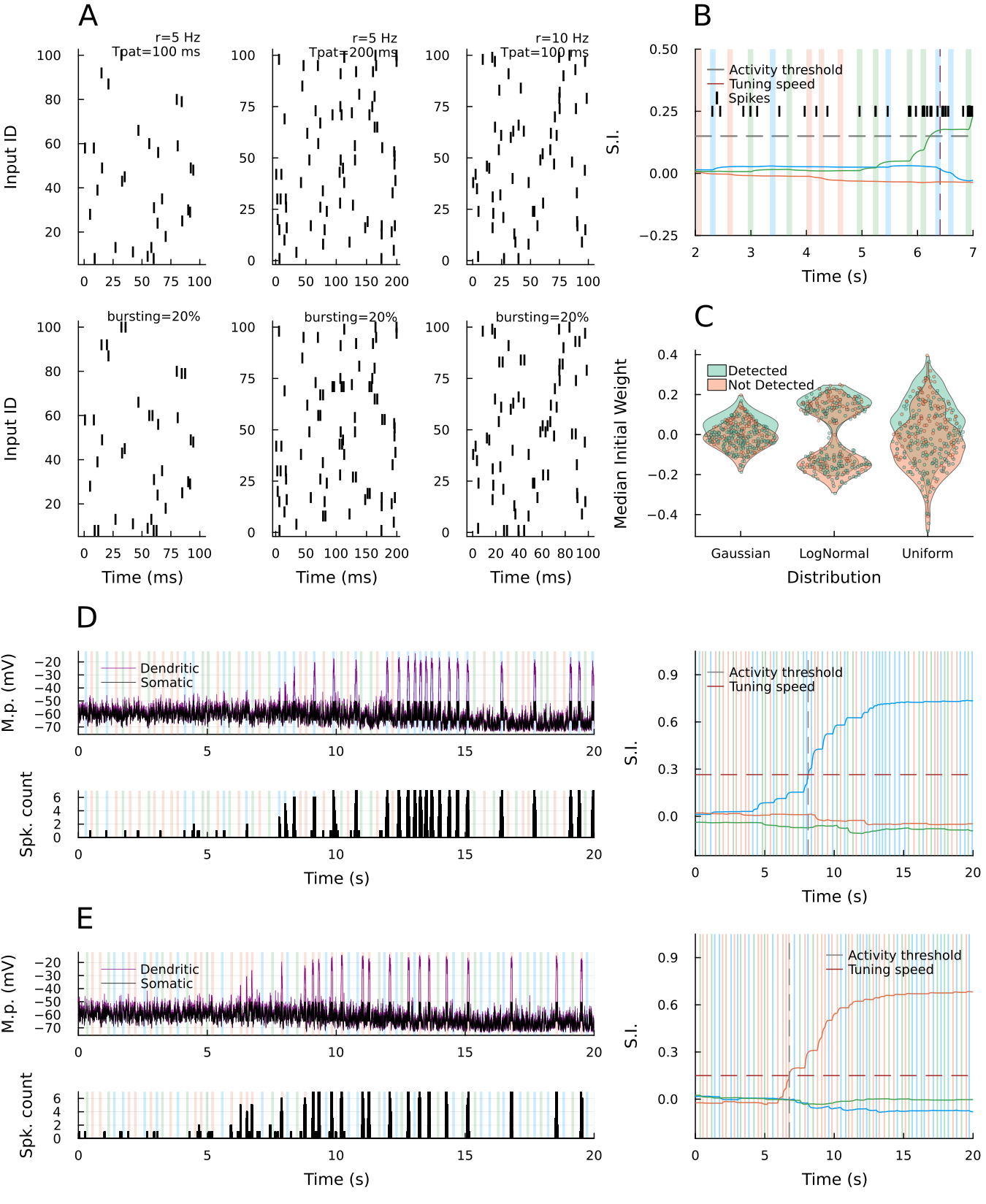

Supplement: S1 Fig — A: Raster plots of three stereotypical patterns generated from Poisson processes. Plots show pattern examples of 100 units spiking for Tpat milliseconds at rate r. Pattern parameters are expressed in text on the top row. Bottom row are raster plots with 20% of units bursting. Bursting units are picked randomly. B: Selectivity index computed across time for an example random neuron simulation trial. The index of each pattern changes across time and the tuning speed (in seconds; brown dashed line) is obtained when the activity reaches the threshold (gray dashed line). C: Median initial synaptic weight value for detected (tuned) and non-detected (no tuning) patterns at different initial sampling distribution. Gaussian distributed synaptic weight values yield the least amount of bias. D: Simulation trial example showing, on the right, the dendritic and somatic compartment membrane potential traces (purple, and black respectively; top row), and somatic peristimulus time histogram (bottom) showing the spike response of the model to the learned pattern (blue). On the right, the selectivity index computed across time for the given trial, and tuning speed (in seconds) is obtained when conditions are met. E: Same as in D, but the input pattern now has 10% of its units bursting across time, decreasing the time it takes to develop a robust response (tuning time). (TIFF) [file pcbi.1013777.s001.tiff]

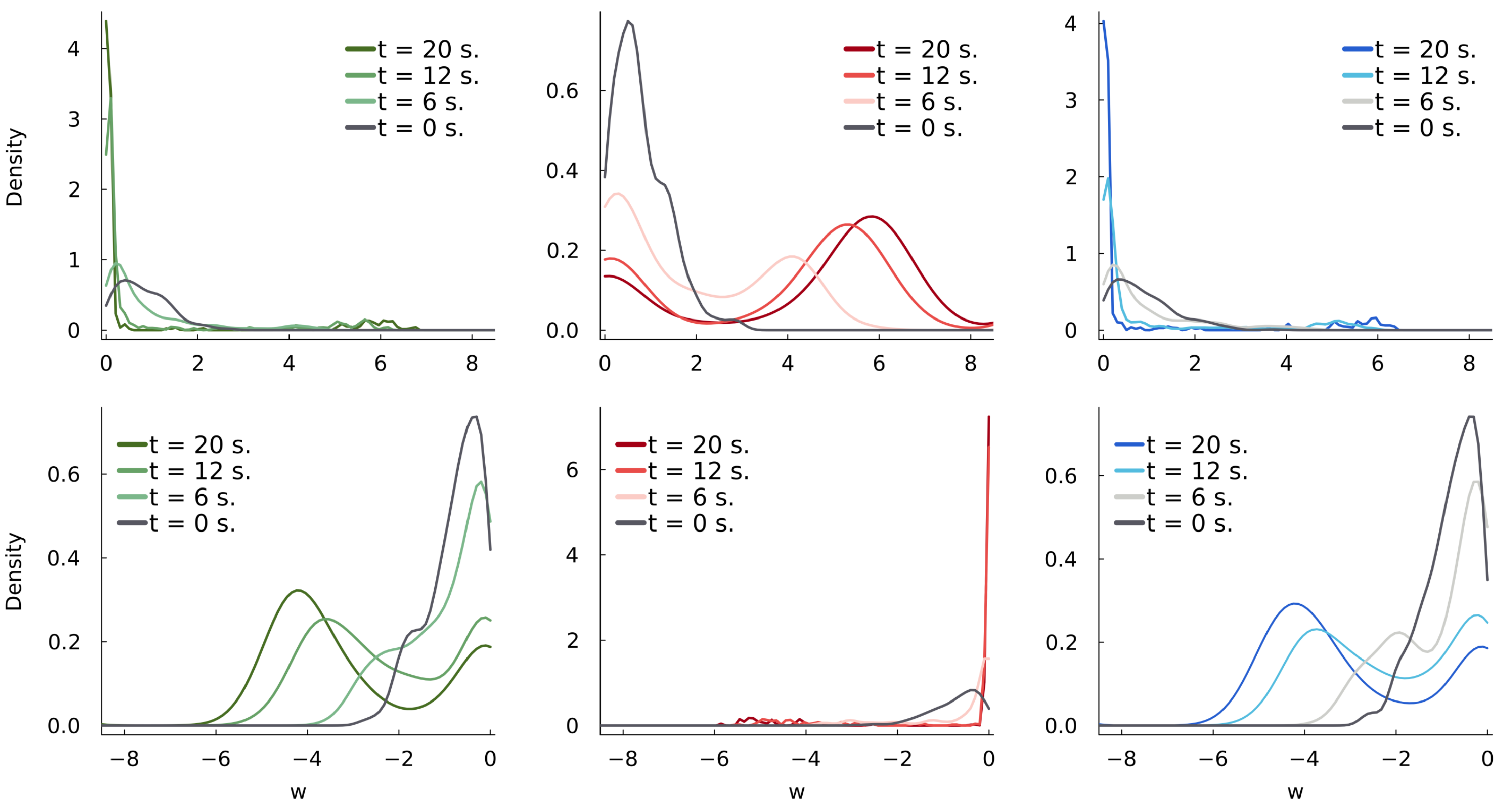

Supplement: S2 Fig — Colored lines from estimations at t=0,6,12,20 seconds of a single trial. Top row: Distributions for excitatory synapses pooled for red, green, and blue patterns. Across time, red pattern pooled excitatory synapses become bimodal with a large amount of strong synapses. Bottom row: Distributions for inhibitory synapses pooled for red, green, and blue patterns. Across time, blue and green pooled inhibitory synapses become bimodal exhibiting strong inhibition with blue and green pattern presentations relative to red pooled inhibitory synapses. Selectivity to a single pattern is hinted as both dense excitatory links for red pattern as well as dense inhibitory links for green and blue pooled synapses. (TIFF) [file pcbi.1013777.s002.tiff]

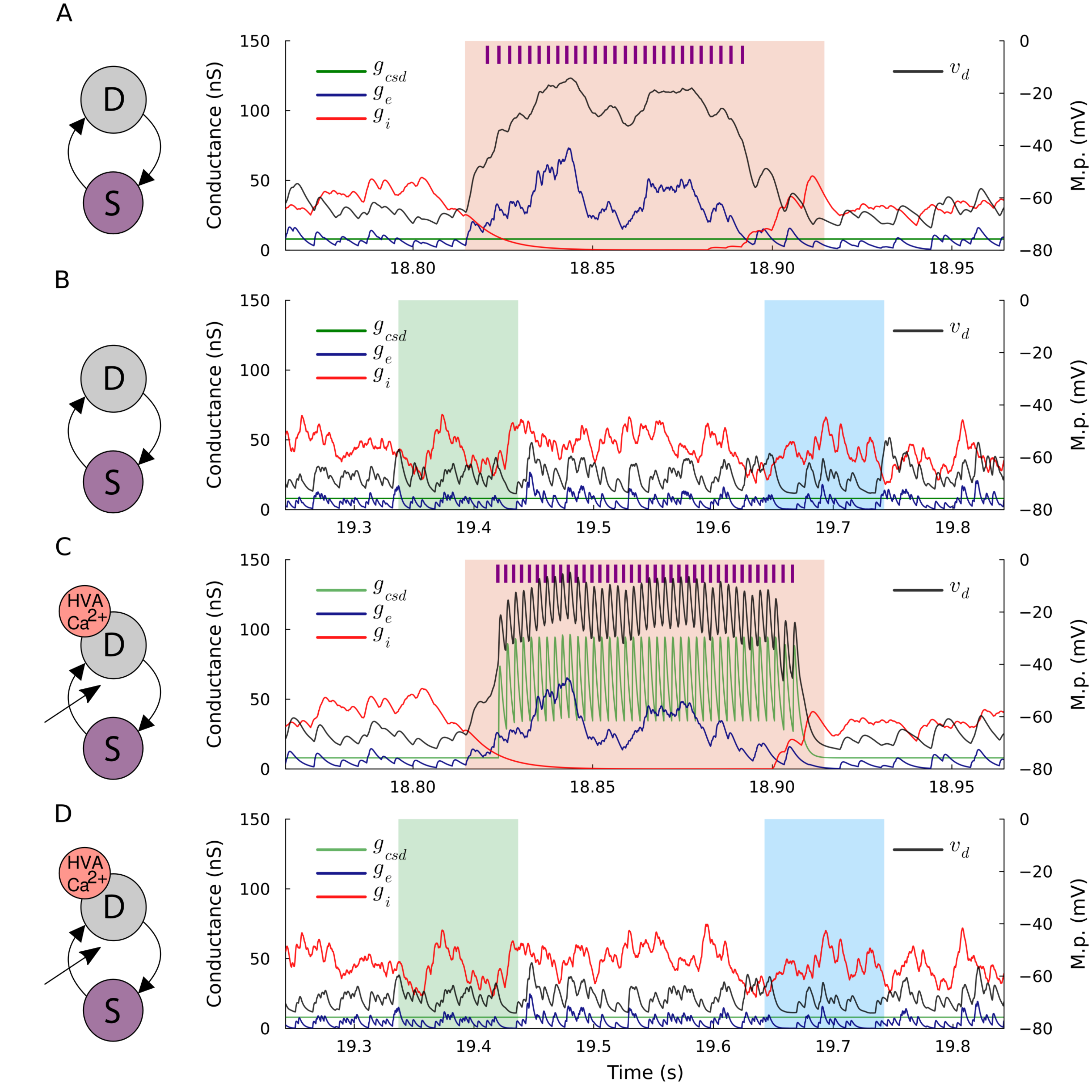

Supplement: S3 Fig — The corresponding model layout on the left of each time course plot. Red pattern tuned trial. A: Spike trace-based model conductances (blue: excitatory, red: inhibitory, green: somato-dendritic) and dendritic membrane trace (black; axis on the right) during red pattern presentation at the end of the simulation. Vertical lines on top represent model output spikes. Inhibitory conductance plummets as an effect of learning while excitatory conductance remains high during red pattern presentation. B: Same as above but for green and blue pattern presentations. Inhibitory conductance is high relative to excitatory conductance. C: Same as in A but for calcium-based model conductances. gcsd is transiently boosted on each somatic spike to enhance dendritic membrane potential peaks. D: Same as B but for the calcium-based model. (TIFF) [file pcbi.1013777.s003.tiff]

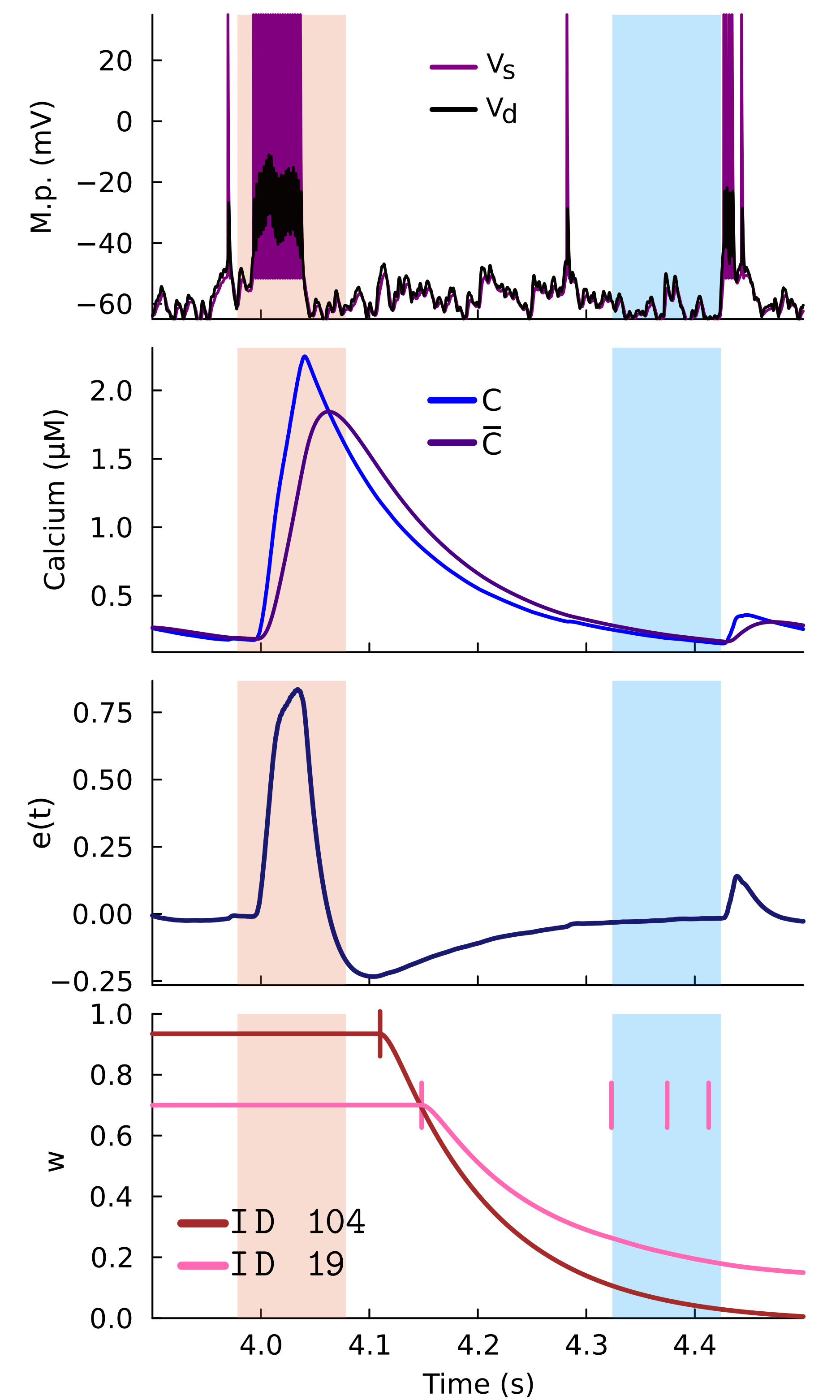

Supplement: S4 Fig — First panel: Membrane potential traces (left y-axis) of somatic (purple) and dendritic (gray) compartments. Second panel: Calcium C(t) (blue) and low-pass filtered C(t)― (midnight blue) traces. Third panel: Plasticity induction error signal e(t). Fourth panel: Synaptic weight changes Δw of input IDs #109 (scarlet) and #19 (pink) traces across time. Vertical marker indicating spike time of input. Strong potentiation is followed by a long-lasting LTD phase yielding a negative phase of e(t) after the preferred pattern. Note that spikes of ID #19 (pink) occurring during the blue pattern do not affect plasticity induction of w19 as the sample of e(t) at given spike times is low (trend remains the same). (TIFF) [file pcbi.1013777.s004.tiff]

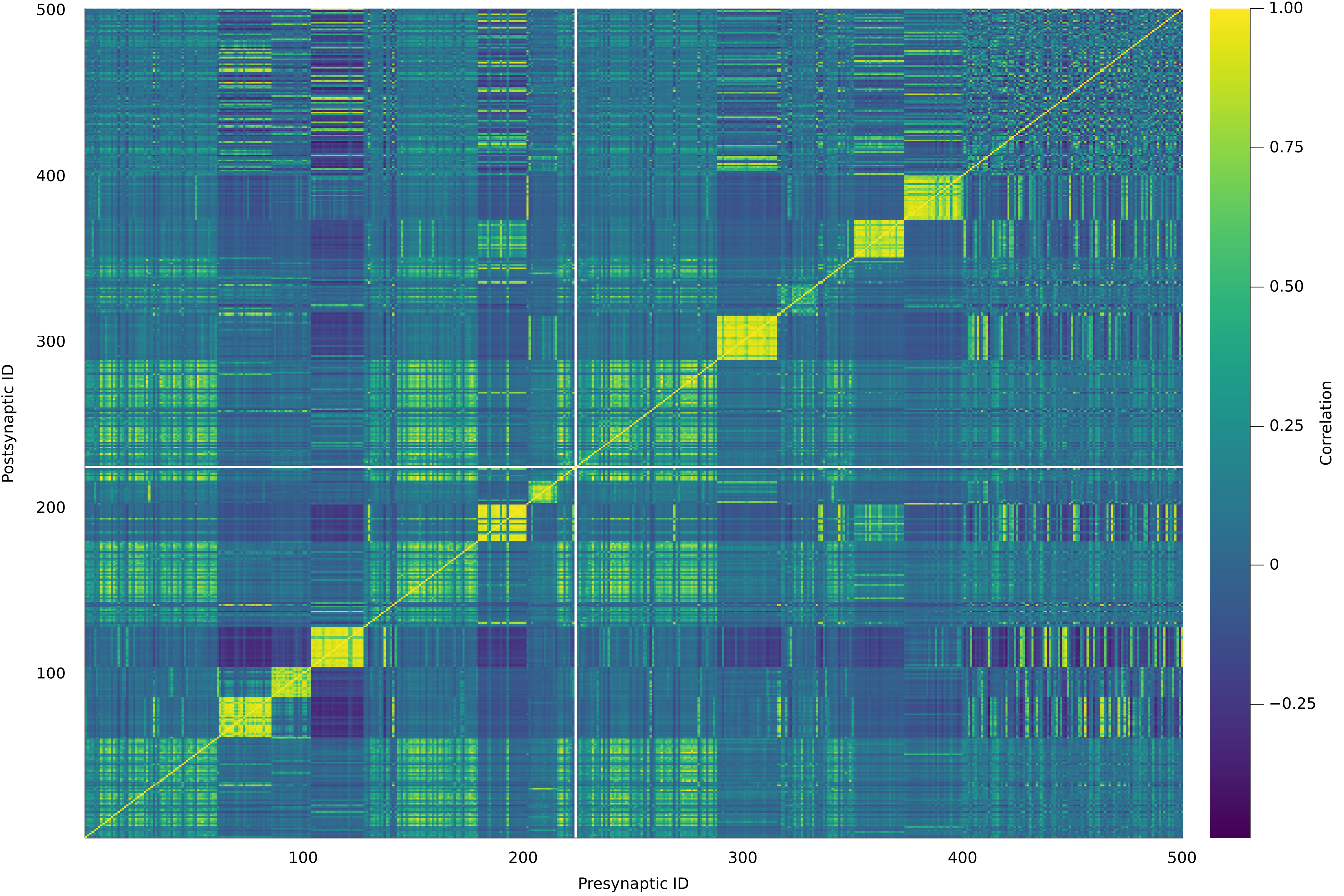

Supplement: S5 Fig — Each entry represents the Pearson correlation between the binned spike trains of two neurons (25 ms bin width). Distinct blocks of elevated correlation emerge along the diagonal, corresponding to the predefined assemblies and confirming temporally coordinated spiking within these groups. Low correlations outside these blocks reflect weak synchrony between unrelated neurons. The white line displays neurons which exhibited no spiking activity during the simulation, leading to undefined correlations (NaN). The matrix demonstrates that structured intra-assembly connectivity results in reliably clustered spiking activity at the network level. (TIFF) [file pcbi.1013777.s005.tiff]
