# Supplementary material for: Transient boosting of action potential backpropagation for few-shot temporal pattern learning
Source: PLoS Comput Biol. 2025 Dec 5;21(12):e1013777. doi: 10.1371/journal.pcbi.1013777 (PMC12698000; doi:10.1371/journal.pcbi.1013777)
Supplement: S1 Table — (PDF) [file pcbi.1013777.s006.pdf]

S1 Table Membrane and synaptic parameter values across models.

Symbol and Description		Spike-based	Calcium-based	Ca+NMDA	Units
g_{cds}	Dendro-somatic conductance	108.0	108.0	108.0	nS
g_{csd}	Somato-dendritic conductance	8.0	Eq 29	Eq 29	nS
g_{csdr}	Somato-dendritic resting conductance	—	8.0	8.0	nS
g_{ls}	Somatic leakage conductance	12.0	12.0	12.0	nS
g_{lsr}	Somatic leakage conductance (refractory)	—	150.0	150.0	nS
g_{ld}	Dendritic leakage conductance	10.0	10.0	10.0	nS
$\overline{g_e}$	Excitatory (AMPA) peak conductance	0.5	0.5	0.5	nS
$\overline{g_i}$	Inhibitory (GABA) peak conductance	0.55	0.55	0.55	nS
$\overline{g_{Ca}}$	HVA-Ca^{2+} peak conductance	—	0.01	0.01	nS
$\overline{g_{NMDA}}$	NMDA receptor peak conductance	—	—	0.0005	nS
C_s	Somatic capacitance	180.0	180.0	180.0	pF
C_d	Dendritic capacitance	60.0	60.0	60.0	pF
V_{th}	Membrane threshold potential	−50.0	−50.0	−50.0	mV
V_{peak}	Membrane peak potential	—	35.0	35.0	mV
V_{re}	Membrane reset potential	−60.0	—	—	mV
V_{ls}	Somatic resting potential	−70.0	−70.0	−70.0	mV
V_{ld}	Dendritic resting potential	−70.0	−70.0	−70.0	mV
V_{er}	Excitatory reversal potential	0.0	0.0	0.0	mV
V_{ir}	Inhibitory reversal potential	−75.0	−75.0	−75.0	mV
V_{Ca}	Ca^{2+} reversal potential	—	120.0	120.0	mV
V_{NMDA}	NMDA reversal potential	—	—	0.0	mV
$\tau_{e,rise}$	Excitatory synapse rise time	0.5	0.5	0.5	ms
$\tau_{e,decay}$	Excitatory synapse decay time	3.0	3.0	3.0	ms
$\tau_{i,rise}$	Inhibitory synapse rise time	1.0	1.0	1.0	ms
$\tau_{i,decay}$	Inhibitory synapse decay time	8.0	8.0	8.0	ms
$\tau_{nmda,rise}$	NMDA rise time	—	—	3.3	ms
$\tau_{nmda,decay}$	NMDA decay time	—	—	102.38	ms
$\tau_{csd,rise}$	g_{csd} boost rise time	—	0.2	0.2	ms
$\tau_{csd,decay}$	g_{csd} boost decay time	—	1.5	1.5	ms
f	NMDA-to-I_{Ca} current factor	—	—	0.05	unitless
