# Supplementary material for: Transient boosting of action potential backpropagation for few-shot temporal pattern learning
Source: PLoS Comput Biol. 2025 Dec 5;21(12):e1013777. doi: 10.1371/journal.pcbi.1013777 (PMC12698000; doi:10.1371/journal.pcbi.1013777)
Supplement: S2 Table — (PDF) [file pcbi.1013777.s007.pdf]

S2 Table Synaptic plasticity rule parameter symbols, values, and descriptions.

Symbol and Description		Spike-based	Calcium-based	Ca+NMDA model	Units
C_0	Baseline Ca^{2+} concentration	—	$50e^{-6}$	$50e^{-6}$	mM
ϕ	I_{Ca} to Ca^{2+} scaling	—	0.15	0.15	mM/pA
τ_Y	Graded spike trace time constant	100.0	—	—	ms
$\tau_{\overline{Y}}$	LPF spike train trace constant	20.0	—	—	ms
τ_Δ	Plasticity induction LPF constant	100.0	100.0	100.0	ms
τ_p	Synaptic displacement time constant	10.0	10.0	10.0	ms
T_κ	Synaptic renormalization interval	1.0	1.0	1.0	ms
ζ_ϑ	ζ-function threshold constant	$1.0e^{-4}$	$1.0e^{-4}$	$1.0e^{-4}$	unitless
τ_ζ	ζ-function time constant	0.75	0.75	0.75	ms
η_{eff}	Effective learning rate	$5e^{-6}$	$5e^{-6}$	$5e^{-6}$	$\eta\delta t^2/\tau_\Delta$
m_{csd}	bAP strength of g_{csd}	—	60.0	60.0	nS
τ_C	Ca^{2+} LPF time constant	—	100.0	100.0	ms
$\tau_{\overline{C}}$	LPF Ca^{2+} trace time constant	—	20.0	20.0	ms
p_{fail}	Synaptic failure probability	0.3	0.3	0.3	unitless
κ^*	Synaptic rescaling parameter	3.5	3.5	3.5	unitless
ρ	STDP induction noise parameter	—	2^{-4}	—	mM

Plasticity rules vary by model type, incorporating either spike timing, intracellular calcium, or NMDA-gated calcium current influence.
