# Supplementary material for: Transient boosting of action potential backpropagation for few-shot temporal pattern learning
Source: PLoS Comput Biol. 2025 Dec 5;21(12):e1013777. doi: 10.1371/journal.pcbi.1013777 (PMC12698000; doi:10.1371/journal.pcbi.1013777)
Supplement: S3 Table — (PDF) [file pcbi.1013777.s008.pdf]

S3 Table Recurrent network with assemblies: parameter symbols, values, and descriptions.

Symbol and Description		Parameter Value	Units / Notes
C_x	Somatic membrane capacitance	exc=180, inh=150	pF
N_x	Number of recurrent units	exc=400, inh=100	units
$p_{e\to e}$	Exc.-Exc. connection probability	0.1	unitless
$p_{e\to i}$	Exc.-Inh. connection probability	0.3	unitless
$p_{i\to e}$	Inh.-Exc. connection probability	0.4	unitless
$p_{i\to i}$	Inh.-Inh. connection probability	0.5	unitless
$W_{e\to e}$	Exc.-Exc. synaptic weight distribution	LogNormal(-0.42, 0.79)	mean, std
$W_{e\to i}$	Exc.-Inh. synaptic weight distribution	LogNormal(0.48, 0.63)	mean, std
$W_{i\to e}$	Inh.-Exc. synaptic weight distribution	LogNormal(-0.74, 0.96)	mean, std
$W_{i\to i}$	Inh.-Inh. synaptic weight distribution	LogNormal(-0.39, 0.78)	mean, std
p_{cl}	Assembly unit connection probability	0.5	unitless
n_{cl}	Assembly size distribution	Normal(18.0, 3.0)	integer (mean, std)
Strong W_{cl}	Strong assembly weight distribution	Normal(3.0, 1.0)	mean, std
Weak W_{cl}	Weak assembly weight distribution	Normal(0.0, 1.0)	mean, std
η_{aff}	Learning rate for afferent inputs	0.05	unitless
η_{rec}	Learning rate for recurrent inputs	0.05	unitless
η_{eff}	Effective learning rate	$5e^{-6}$	$\eta \delta t^2/\tau_\Delta$

Most cell parameters as in the spike trace-based model, unless otherwise noted. Assembly-specific synapses were divided into strong and weak distributions.
