# Supplementary material for: Transient boosting of action potential backpropagation for few-shot temporal pattern learning
Source: PLoS Comput Biol. 2025 Dec 5;21(12):e1013777. doi: 10.1371/journal.pcbi.1013777 (PMC12698000; doi:10.1371/journal.pcbi.1013777)
Supplement: Attachment — Submitted filename: review_v1.pdf [file pcbi.1013777.s010.pdf]

In their paper “Transient boosting of action potential back propagation for few-shot temporal pattern learning”, Sivori & Fukai propose a novel “self-supervised” learning rule that enables neuron models to detect recurring patterns of input spikes, without specific supervisory signal. To achieve this, the rule combines the presynaptic input, in the form of a PSP term, a weight scaling term to avoid runaway effects and match a reasonable spine distribution, and an ‘excitability signal’. The latter corresponds most closely to the credit assignment terms found in many learning rules, but instead of a target signal, if features a type of high-pass filter of the output spike train, construct as the difference of a low pass filter of the output spikes, and another low pass filter of this low pass filter. Consequently, this means that transient increases in spike rate will lead to positive changes in synaptic weights, while transient decreases will lead to negative changes in synaptic weights. Notably, the authors show that this excitability signal is sufficient to learn input spike patterns amidst synaptic transmission failures, even when a only single output spike is generated initially.
Crucially, the authors demonstrate that this credit assignment signal, or ‘excitability signal’ as they call it, can be implemented by biophysically plausible calcium dynamics. They demonstrate this by implementing a voltage-activated calcium channel that changes the calcium concentration, and they show that their learning rule also works with this concentration signal. This is a very interesting aspect of the paper, as the literature that I am aware of does not take this step of showing how the biophysics can successfully lead to this credit assignment signal. While overall I therefore think this work is very relevant and interesting, there are some assumptions in the model that merit some stronger motivation, as I’ll explain in my detailed comments below.

Major comments

1. My main concern pertains to the time-varying nature of the somato-dendritic coupling conductance. The learning rule proposed by the authors depends on an increase in calcium concentration in the dendritic compartment, which requires backpropagation of the AP. In neurons, this is thought to be governed by a density of sodium channels distributed on the apical trunk, which can clearly not be reproduced by the same mechanism in the two-compartment model, as all that is available in this simplified model is a passive coupling conductance between two compartments. Although I do not reject the possibility that the effective coupling between compartments may change due to active currents, I wonder whether the assumption described in the paper (page 38-39) are correct, as I would expect the coupling conductance to decrease as more membrane conductance are open (for instance due the the opening of potassium currents to repolarize the dendrite after the AP has passed). In my view, the authors should better motivate this critical assumption in their model (note that the concerned section of text doesn’t cite any references either).
2. Related to point 1, I wonder why the authors did not choose a simpler mechanisms to get the backpropagating AP in the dendritic compartment, such as the injection of a current waveform triggered by the somatic AP, as implemented e.g. in Pastorelli et al. (https://doi.org/10.48550/arXiv.2311.06074)?
3. My final major comment pertains to the spike-rate after learning. It appears that once a pattern has been learned, the firing rate is extremely high during the presentation of the pattern, with the neuron(s) spiking nearly continuously during the presentation. This does not appear physiological. Is there a way to tune the model, such that a more physiological firing rate is reached after pattern learning?

Minor comments:

1. On page 2: “At least, two different computational approaches can be made to pattern detection in neurons. In one approach, synaptic learning rules derived from experimental evidence such as spike-timing- dependent plasticity (STDP) are examined for the purpose.” These sentence could benefit from an improved formulation (e.g. “can be made” -> “exist in the literature”, “are examined” -> “are investigated”.
2. On page 2: “The latter tends to perform well on various tasks as the underlying learning rules are designed to optimize their performance in learning” This reads like a circular reasoning.
3. On page 3: “well-based”. Do the authors mean “well defined”?
4. On page 5: “…for these and similar approaches, including ours…” -> formulation could be simplified.
5. On page 5: “… 2) postsynaptic potentials (PSPs) evoked by presynaptic spikes (Fig. 1B),…” figure 1B doesn’t show PSPs.

6. On page 13: ref to Fig 3B and 3D -> should be supplementary Fig 3B and 3D, I presume.
7. On page 16: “For this case, we observe that the inclusion of NMDA receptor dynamics in the dendritic compartment can result in steeper rising and falling edges of the excitability signal $e_C(t)$, which now captures many more synapses during post-synaptic spiking.” -> Can this be observed somewhere?
8. Fig 3C: Perhaps it would be clearer to stack the bar plots for each configuration.
9. In the recurrent network (page 24), do the recurrent connections arrive at the soma or dendrite?
10. Page 30: “…bursts are thought to transmit spikes more faithfully than single spikes.” This formulation is not clear.
