# Supplementary material for: Transient boosting of action potential backpropagation for few-shot temporal pattern learning
Source: PLoS Comput Biol. 2025 Dec 5;21(12):e1013777. doi: 10.1371/journal.pcbi.1013777 (PMC12698000; doi:10.1371/journal.pcbi.1013777)
Supplement: Attachment — Submitted filename: Reply to Reviewers.pdf [file pcbi.1013777.s011.pdf]

Manuscript #: PCOMPBIOL-D-24-01766
Title: Transient Boosting of Action Potential Backpropagation for Few-shot Temporal Pattern Learning
Authors: Gaston Sivori and Tomoki Fukai

We thank the reviewers for their overall positive comments on our manuscript. Following their comments, we have revised our manuscript, including novel simulation results. Below is our point-to-point reply in bold style. The reviewers' comments are shown with regular fonts. We hope that our revisions clarify all the concerns raised by the reviewers.

Reviewer #1:
Summary:
The paper introduces a self-supervised plasticity rule for few-shot pattern learning. The plasticity rule consists of three parts in a two-compartment neuron model: the self-supervised error signal resembles the sliding window of BCM-rule except with eligibility trace of spike trains; the post-synaptic potential is eligibility trace of total input; the scaling term is a boundary factor for synaptic weight.
The original form is extended to a calcium-based form where the concentration of calcium serves as the substrate for eligibility traces and the error signal is mediated by bAP, i.e., somato-dendritic coupling. The calcium-based form is biologically plausible, and the simulations fit with single-doublet pairing protocol induced Schaffer-collateral STDP in hippocampus. The performance of pattern learning is further improved by the insertion of NMDARs which promotes bursting activities.
Lastly, the paper proposes a recurrent scheme with preconfigured small assemblies. The original form efficiently learns the input pattern with just several shots by recruiting the pre-existing assemblies.
The paper in general did solid modeling and simulations. The idea of using calcium as the substrate for eligibility trace is interesting. However, there are some subtle assumptions that need further checking, especially the motivation for the complexity of models. Next page are the major comments regarding these assumptions and model choice.
In the last page, I have some minor comment line by line.

We thank the reviewer for the valuable comments. Please find our point-to-point reply below.
Major comments:

1. In all settings, the feedforward(input) connections are all-to-all. This would nevertheless induce some level of initial bias in network response, e.g., some neurons would have higher response to certain input patterns. How strong is this initial bias in your model? In addition, how sensitive is performance to initialization of weights? (e.g., instead of gaussian, log-normal or uniform)

   To assess whether the initialization of synaptic weights introduces bias in detection outcomes, we ran simulations using three distinct distributions: Gaussian, Uniform, and LogNormal. For the Gaussian distribution, the weights were drawn from a standard normal distribution ($\mu = 0$, $\sigma = 1$), allowing for both positive and negative values symmetrically. To ensure fair comparisons, we selected the bounds of the Uniform distribution as $[-3, 3]$, which matches the approximate 6-σ (99.7%) support range of the Gaussian. For the LogNormal distribution, we used parameter values $\mu = -0.3466$, $\sigma = 0.8326$, yielding a distribution with approximately the same variance

as the Gaussian (i.e., $\mu \approx 1$, $\sigma \approx 1$). As LogNormal is strictly positive, inhibitory synaptic weights were just multiplied by -1 to make the overall mean zero. Matching the standard deviation gives us a fair comparison of spread, and choosing parameters to yield $\mu \approx 1$ ensures the typical weight magnitudes are comparable—even if their distributions are not.

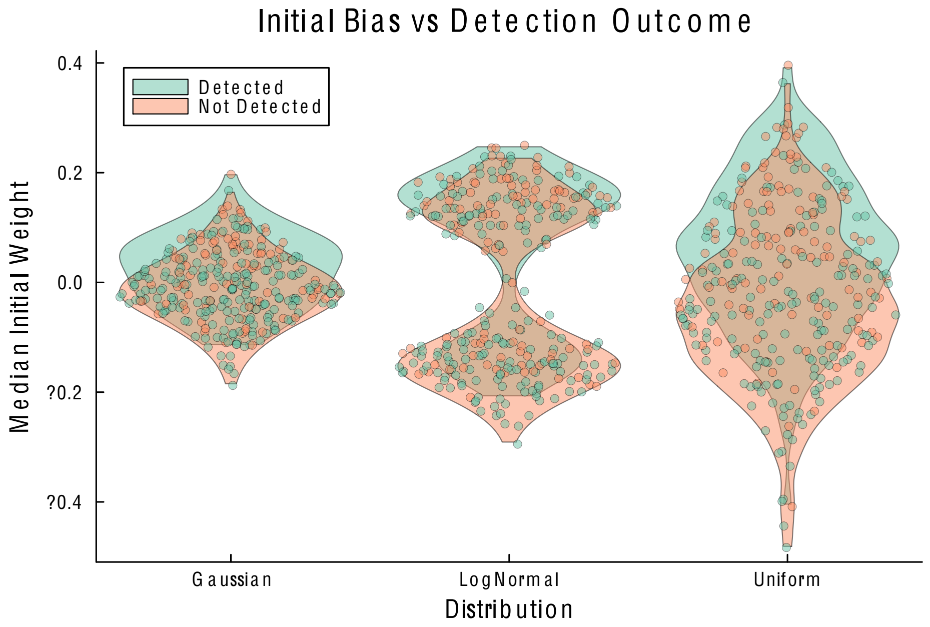

As shown above in our violin and boxplot analyses, all three weight distributions exhibit statistically significant differences in initial median weights between detected and non-detected patterns (Mann–Whitney U tests, all $p < 10^{-4}$). This suggests that the initial bias in weight values can influence early pattern selection, even in randomized all-to-all settings. Namely, an input pattern mediated by stronger initial weights is more likely to be detected.

It is worth noting that in our model, each synapse has a 30% probability of transmission failure per spike. This unreliability introduces substantial stochastic variability in synaptic input, which likely reduces the impact of initial weights on postsynaptic firing in any given trial (see manuscript Subfigure 1 for examples). Thus, the observed statistical bias may reflect the net effect of both the initial weight distribution and synaptic-level noise. We have clarified this point in the revised manuscript, as specified below, and included this subfigure as Supplementary Fig. 1C.

Changes in the manuscript:
- **Revised main text: Page 8, line 10, paragraph 2.**
- **New supplementary subfigure 1C. Revised the subsequent subfigure labels.**
- **New methods section for further details: "Initial bias sensitivity analysis".**

2. The homeostasis has extremely fast updating (time constant of 1.0ms), this does not align with experiments that homeostasis is usually of a much longer time scale.

We assume that the reviewer's concern is about the smallness of the variable T_κ (there was a typo in the previous manuscript: $T_\theta \to T_\kappa$). We appreciate the reviewer's concern,

but we would like to clarify that the variable is not meant to represent a biophysical homeostatic mechanism with a biological timescale (i.e., intrinsic plasticity). Instead, it renormalizes synaptic weights (i.e. synaptic scaling) at the fixed update interval T_κ. This interval only affects the granularity of κ in numerical simulations but does not model any physical time constant. To illustrate this point, we ran a single simulation at different update interval values: T_κ = 1, 10, 100, and 1000 milliseconds. Despite this wide range of update frequencies, the evolution of κ over time remains largely unaffected in terms of its overall decay profile — indicating that the underlying process is driven by network activity and weight changes, not the frequency of update checks. We have clarified this point in the revised Methods section now renamed "Synaptic rescaling". We have also modified Eq. 34 as the previous expression of update rule for κ was misleading.

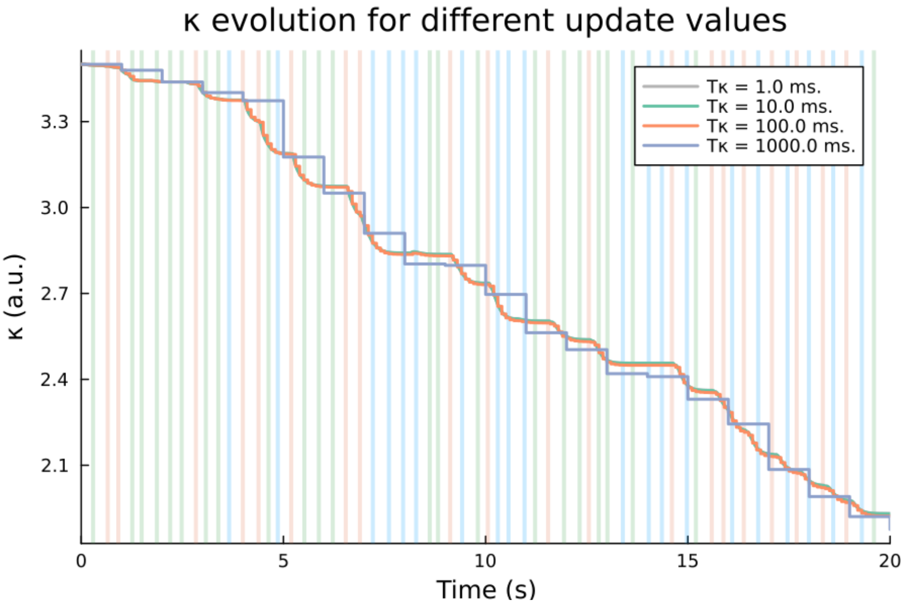

3. In eq.18, an extra low-pass filter (time constant 100ms) is used for PI(i,t). Is this necessary as PI(i,t) itself is the product of filtered signals e(t) and PSP(i,t)?

   We appreciate the concern. While e(t) and PSP(i,t) are indeed filtered individually, we apply an additional low-pass filter to this product to ensure stable learning dynamics. Without this filtering step, the product PI(i,t) can undergo sudden large increases, and this results in abrupt and destabilizing synaptic weight changes Δw_i since they directly integrate this value. The extra low-pass filter thus ensures that weight updates occur gradually over time preventing runaway excitation or numerical instability.

4. In the Ca-NMDARs model, the AMPA/NMDA ratio is chosen as 5%. Is there experimental support or just to have minimal effect on dend depolarization? In addition, the effect of NMDARs is to promote bursting. What is the difference between NMDARs insertion and explicitly setting 10% input neurons to burst?

   We appreciate this comment as well. There might be a confusion about the parameter $f = 5\,\%$. This parameter does not represent AMPA/NMDA ratio at excitatory synapses but rather represents the ratio of calcium current to the total (i.e., glutamatergic plus

calcium) NMDA receptor-mediated current under physiological conditions. This percentage is typically assumed between 5-10% (e.g. Schiller et al. 2000) based on experimental observations at typical external calcium concentrations of 1~2mM (Jahr & Stevens, 1993). We have clarified this point on page 16, paragraph 1, and included the reference above for completeness.

We noticed that the AMPA/NMDA ratio chosen for the Ca-NMDA model was not reported in the previous manuscript. As not all synapses tend to include AMPA, we chose a ratio of around 75%, the values typically found in Schaffer Collateral synapses in CA1. We have clarified this point in the revised manuscript and included two relevant references.

Regarding the promotion of bursting, NMDARs provide sustained depolarization due to the longer decay time. NMDAR insertion allows the calcium-based model to rely on this timescale to further increase the signal-to-noise ratio of detected input patterns. In contrast, the explicit setting of bursting inputs does not induce the slow intracellular process and does not increase the SN ratio. With the exception of the tests in the Subfigure 1, we did not include bursting units as this scenario is out of the scope of this work. However, in the cortical network simulations, bursting recurrent inputs from cell assemblies may accelerate a convergence to input pattern detection.

5. There is no mentioning of synaptic delay in either the single neuron or network model. How is it defined? Would synaptic delay change your results?

   We thank the reviewer for this comment. Indeed, our models do not include any synaptic delay; all synaptic effects are assumed to be instantaneous relative to the simulation timestep. This was adopted to focus on the core plasticity dynamics and tuning behavior, which occur over timescales much slower than typical synaptic delays. Therefore, we do not expect that biologically realistic synaptic delays (typically 0.5~2ms) affect our essential results, as the learning rule integrates over extended time windows and is not critically dependent on precise spike timing. We have explained these points on page 7, paragraph 3.

6. In the recurrent network model, the connectivity is set to a scheme where inhibition is strongly recruited by excitatory neurons (Exc-Inh is very strong). It looks like a trivial choice. Is this a necessary condition?

   The reviewer pointed out an important point. The relatively strong Exc-Inh connectivity which is a common motif in cortical circuits, ensures that inhibitory neurons are robustly activated by excitatory activity. This plays a critical role in establishing an initial excitation-inhibition (E/I) balance necessary for preventing runaway excitation and facilitating stable network dynamics at the onset of simulations.
   In addition, while the initial connectivity supports strong balance, we emphasize that it is not fixed: the plasticity rule continuously adjusts both excitatory and inhibitory weights. Thus, the network self-organizes into a novel balanced, pattern-driven configuration. In this context, the initial EI bias serves as a scaffold rather than a fixed constraint, and

plasticity ensures that any functional imbalance is corrected during learning. These points are mentioned on page 25, paragraph 2.

7. The authors argue that the pre-existing assemblies provide initially active neurons. What about the pre-existing within-assembly connectivity? Would random connectivity with some neurons being intrinsically more active give similar results?

   We greatly appreciate this interesting question. As shown in the violin plots discussed previously, the initial bias of synaptic weights favors certain postsynaptic neurons to tune more rapidly to a pattern than others. Once a neuron starts to tune to a patterned input, pre-existing assemblies facilitate rapid tuning by amplifying the activity of early spikers. In this way, pre-existing assemblies shape network dynamics and contribute to faster convergence. Such a feedback effect should arise for random recurrent connectivity, and we should observe similar results. However, we speculate that the learning speed is not as high as in networks with pre-existing cell assemblies, but somewhat higher than in single-cell-only settings. Due to time limitations, we have not quantitatively addressed this point in the revised manuscript.

8. A general concern is there are so many parameters hence different combinations could lead to the same results(redundancy). How do you argue for the choice of conductance and time constants? More, specifically how do you justify the relative value of different conductance and time constants?

   We appreciate the reviewer's concern regarding parameter choice and potential redundancy. All conductance values and time constants were chosen to reflect their distinct roles in the corresponding mechanisms within the ranges commonly reported in experimental studies. Importantly, the model's qualitative behavior—particularly the emergence of pattern-specific tuning—is robust across physiological ranges of conductance and time constant values, provided that E/I balance is maintained. Therefore, the values can be modified if needed. We did not aim to fit the model to specific datasets but rather to reproduce biologically plausible dynamics under grounded assumptions. Indeed, small variations in parameter values yielded similar outcomes and, accordingly, we originally opted not to include individual citations for each parameter. To amend this, in the revised manuscript, we have included a subsection in Methods titled "Synaptic and intrinsic parameter selection". In this subsection, we discussed our rationale and cited relevant literature.

Detailed comments:

1) Page 6 Eq. 17, the Alpha function gives rise to typical spine head volume distribution under what condition?

   The proposed Alpha function is meant to generate synaptic weight distribution that matches physiological spine head volume distribution under spontaneous activity as observed in experimental data (Yasumatsu et al. (2008): cultured cell slices from hippocampal CA1: see the comparison below).

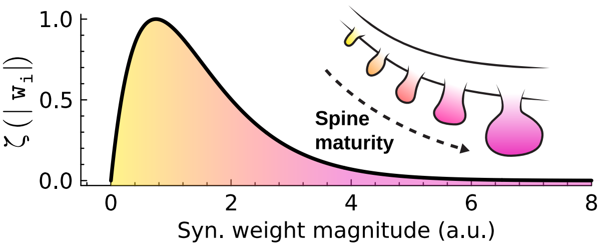

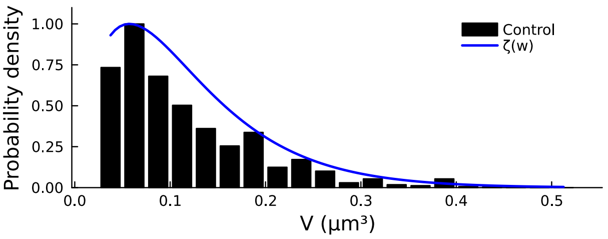

We realized that this important piece of information was missing in the manuscript. The Alpha function can bound synaptic weights dynamically through learning but it's not meant to indicate pattern-tuning capacity; rather, it constraints the growth such that small synaptic weights have greater chance to increase and larger weights have near zero chance of increasing further. We clarified this point in the revised manuscript on page 6, penultimate paragraph.

2) Page 6 bottom lines, why equal number of exc and inh input neurons?

We chose an equal number of excitatory and inhibitory input neurons to simplify the initial conditions and avoid introducing bias toward excitation or inhibition. We agree that this setting is oversimplified. However, as the essential results would not change, we did not pursue a more realistic setting of synaptic input.

3) Page 7 paragraph 2 last sentence, what is the distribution of early and late onset responses?

Early and late burst responses refer to neuronal responses to a pattern during 0 to 50 ms or 50 to 100 ms after stimulus onset, respectively. We have added the definitions on the first paragraph of page 8 in the revised manuscript.

4) Page 8 paragraph 1, how is the long-tailed strength distribution related to bimodality of trained weights?

Bimodality merely represents that a postsynaptic neuron has learned to respond selectively to a pattern amongst other patterns and hence arises from competition for postsynaptic activity among input patterns (e.g., see Supplm. Fig. 2 where 20-sec simulations for 2000 inputs are shown). Thus, the long-tailed distribution is not a direct consequence of the bimodality. Long tails can be seen when we plot the histogram of all synapses, implying that synapses mediating the learned pattern are distributed toward the long-tail end whereas those mediating other patterns remain relatively weak.

5) Fig1.F, e(t) is always on(non-zero). What happens if there is a period of silence? Would it drastically change weights?

The excitability trace e(t) is dependent on postsynaptic spiking activity S(t). Indeed, in Fig. 1F e(t) is non-zero but the trace itself can become zero (e(t)=0) in periods of quiescence as the graded spike count Y(t) and the low-pass-filtered spike trace $\overline{Y}(t)$ become zero. This is the expected behavior and does not change synaptic weights. We have clarified this point on the last paragraph of page 11.

6) Page 10 paragraph 2 last line, LTP and LTD become equivalent. Is it strictly or roughly equivalent?

We appreciate this comment. Indeed, our statement on the equivalent LTP and LTD effects was misleading. This equivalence only roughly holds in the sense that the temporal average of the signal e(t) should vanish: $\int_0^T e(t)dt \approx 0$**, where t=0 refers to the onset of a target pattern input and *T* is taken sufficiently longer than the post-stimulus relaxation time (see Fig. 1F). We have clarified this point on the last paragraph of page 11.**

7) Page 13 paragraph 2, what is opposite conductance of the somato-dendritic coupling?

We apologize for the misleading statement . The opposite conductance of the somato-dendritic coupling means the dendro-somatic conductance, i.e., the conductance for the opposite direction of information flow. We note that our neuron model assumes a spike-triggered transient boosting of the somato-dendritic, but not dendro-somatic, conductance. We clarified this definition on the last paragraph of page 14.

8) Page 13 second-last line, is it "(Supp Fig. 3B and 3D)"?

Indeed, this is Supplementary Figure 3B and 3D. We appreciate the reviewer for pointing it out. We have revised the text on page 16, paragraph 1.

9) Fig.2D, add units to color bar.

We appreciate this comment. We have amended this figure in the revised manuscript.

10) Fig.3B, the confidence interval of mean estimation? In Fig2.C, the variance seems very large, therefore is the mean estimation meaningful?

We apologize for any confusion prompted by Fig. 3B. In this figure, we show the mean SNR across 500 simulations, with 95% confidence intervals. The SNR improving trend with the bAP strength is consistent and the intervals being relatively similar, supporting the reliability of the mean. In Fig. 2C, while variance is higher due to trial-to-trial variability, the mean shown still reflects meaningful average behavior. We hope this explanation is sufficient.

11) Page 18 paragraph 2, what is the use of synaptic transmission failure? Simply adding more noise to the Poisson spike trains?

The introduction of synaptic transmission failure is not intended to add arbitrary noise but rather to reflect a biologically grounded feature of synaptic transmission in cortical circuits. This stochasticity has been observed experimentally and introduces trial-to-trial variability in synaptic communication. While this variability indeed increases variability over Poisson inputs, functionally, it encourages robustness in the learning mechanism by preventing overfitting to specific spike coincidences and ensuring that tuning emerges from consistent patterns rather than noise artifacts. We have improved the writing of this rationale on page 19, paragraph 2 of the revised manuscript.

12) Page 28 paragraph 1 last line, with or without?

We appreciate this observation from the reviewer. Our approach offers a biologically plausible realization of self-supervised learning that integrates these rules,"without" assuming any predictive coding framework a priori. On page 29, paragraph 1, we have revised the sentence in the manuscript as follows: "Our work, based on self-supervised learning, sits among this class of algorithms while offering a biologically plausible realization, thereby bridging two classes of synaptic plasticity rules that were not developed with a predictive framework in mind."

13) Page 28 paragraph 2, last line, be hinted at?

We thank the reviewer for this comment. We agree that the phrasing was imprecise. Our motivation is to link the transient boosting of coupling with the emergence of dendritic plateau potentials. We have explained this point on the first paragraph of page 30 in the revised manuscript.

14) Fig.6B, the raster plot is a bit messy. What does pair-wise correlation look like?

We apologize the messy raster plot, and thank the reviewer for this suggestion. We have modified the raster plot in Fig. 6B to improve clarity and highlight coordinated spiking activity by displaying a shorter 5-second window (instead of 10 seconds) and reducing the marker size and stroke width. According to the reviewer's suggestion, we also computed the pairwise spike correlation matrix, using binned spike counts (25 ms bins) across the full 10-second simulation. The resulting matrix (heatmap) reveals clear blocks of elevated correlation corresponding to the predefined assemblies, confirming that within-assembly neurons tend to spike in a temporally coordinated fashion. These correlation clusters align well with the known structure of the network and support the tuning dynamics described in the revised manuscript on page 25, last paragraph.
We also observe low to moderate correlations outside these blocks, indicating limited synchrony across unrelated neurons, as expected. To quantify this, we computed the average pairwise correlations and found substantially higher values within assemblies's

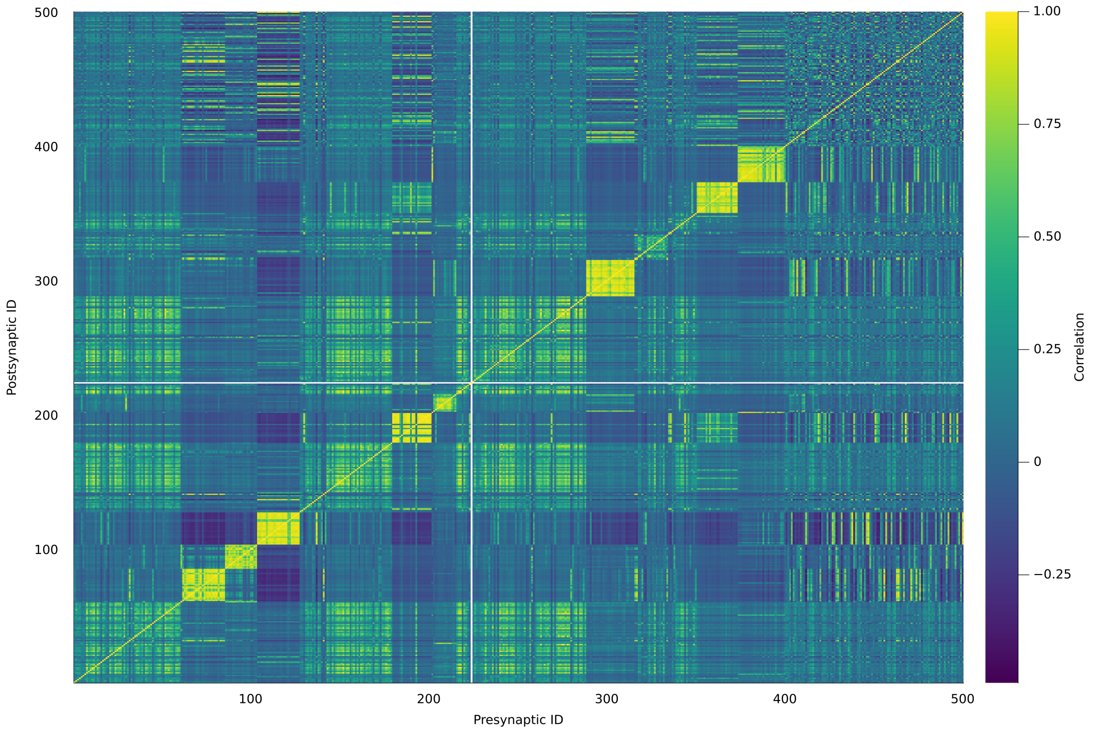

structured activity (0.59) compared to between them (0.09). We have included the result as Supplementary Fig. 5.

15) Fig.6E, does the normalized spike count increase because of decrease of assembly size?

The reviewer pointed out an important point. We have clarified that the observed increase in the normalized spike count is not due to a decrease in assembly size, which remains constant across repeated stimulus presentations. Instead, this increase reflects a more coordinated activation of assemblies per pattern due to gradually strengthened intra-assembly synaptic weights. We have explained this point on page 26, paragraph 2 in the revised manuscript.

16) Page 32 paragraph 2, (but see) what?

We appreciate the comment by the reviewer. We have rewritten the statement to include the reference more clearly on page 33, section "Limitation of the present models".

17) Eq 31, is it Ica (+=) or a different variable Ica+ from Eq 26?

We thank the reviewer for pointing this out. In Eq. 31, we used the notation += following coding conventions (i.e., a += b implies that a+b should be substituted for a) to indicate that the NMDA calcium current from Eq. 26 is added to the HVA calcium current. However, this notation was inadequate, and we have revised the equation in a standard mathematical fashion. To avoid confusion, we have explicitly stated that the total calcium current includes contributions from both the NMDA and HVA-Ca channels (see Methods section "NMDAR dynamics (Ca/NMDAR-based)).

Reviewer #2:

In their paper "Transient boosting of action potential back propagation for few-shot temporal pattern learning", Sivori & Fukai propose a novel "self-supervised" learning rule that enables neuron models to detect recurring patterns of input spikes, without specific supervisory signal. To achieve this, the rule combines the presynaptic input, in the form of a PSP term, a weight scaling term to avoid runaway effects and match a reasonable spine distribution, and an 'excitability signal'. The latter corresponds most closely to the credit assignment terms found in many learning rules, but instead of a target signal, if features a type of high-pass filter of the output spike train, construct as the difference of a low pass filter of the output spikes, and another low pass filter of this low pass filter. Consequently, this means that transient increases in spike rate will lead to positive changes in synaptic weights, while transient decreases will lead to negative changes in synaptic weights. Notably, the authors show that this excitability signal is sufficient to learn input spike patterns amidst synaptic transmission failures, even when a only single output spike is generated initially. Crucially, the authors demonstrate that this credit assignment signal, or 'excitability signal' as they call it, can be implemented by biophysically plausible calcium dynamics. They demonstrate this by implementing a voltage-activated calcium channel that changes the calcium concentration, and they show that their learning rule also works with this concentration

signal. This is a very interesting aspect of the paper, as the literature that I am aware of does not take this step of showing how the biophysics can successfully lead to this credit assignment signal. While overall I therefore think this work is very relevant and interesting, there are some assumptions in the model that merit some stronger motivation, as I'll explain in my detailed comments below.

We thank the reviewer for the thoughtful comments. Please find our point-to-point reply below.

Major comments:
1. My main concern pertains to the time-varying nature of the somato-dendritic coupling conductance. The learning rule proposed by the authors depends on an increase in calcium concentration in the dendritic compartment, which requires backpropagation of the AP. In neurons, this is thought to be governed by a density of sodium channels distributed on the apical trunk, which can clearly not be reproduced by the same mechanism in the two-compartment model, as all that is available in this simplified model is a passive coupling conductance between two compartments. Although I do not reject the possibility that the effective coupling between compartments may change due to active currents, I wonder whether the assumption described in the paper (page 38-39) are correct, as I would expect the coupling conductance to decrease as more membrane conductance are open (for instance due the the opening of potassium currents to repolarize the dendrite after the AP has passed). In my view, the authors should better motivate this critical assumption in their model (note that the concerned section of text doesn't cite any references either).

Thank you for your insightful comment and for raising an important point regarding the time-varying nature of the somato-dendritic coupling conductance in our model.
We agree that the density of sodium channels in the apical trunk plays a crucial role in the backpropagation of action potentials, and this is indeed a key consideration for our model. As the reviewer pointed out, however, in the simplified two-compartment model, we hypothesized that a transient increase in dendritic coupling during bAP events could mimic their effect on the induction of calcium influx. Although the passive coupling between compartments (axial resistance) is certainly a simplification, we believe that this transient increase during bAPs provides a reasonable approximation of the effect of active dendritic conductance.

Regarding your concern that conductance might decrease as the membrane repolarizes, we acknowledge that there are indeed phases where the membrane conductance could be hyper-polarized by active repolarization mechanisms. However, our model focuses on the immediate effect of bAP propagation and its transient boosting driven by calcium influx and other active currents (e.g., sodium spikelets) during the peak depolarization phase. Although the coupling conductance could decrease afterward, our assumption that the initial conductance increase during the bAP event itself is valid for boosting the effective coupling.
According to the reviewer's suggestion, we have revised the manuscript to better motivate the critical assumption, providing relevant references on page 41 in section "Somato-dendritic coupling current (calcium-based)".

2. Related to point 1, I wonder why the authors did not choose a simpler mechanisms to get the backpropagating AP in the dendritic compartment, such as the injection of a current waveform

triggered by the somatic AP, as implemented e.g. in Pastorelli et al. (https://doi.org/10.48550/arXiv.2311.06074)?

Thank you for your suggestion on the use of a current injection waveform to model the backpropagating action potential (bAP) in the dendritic compartment. We were unaware of the method shown in Pastorelli et al. (2023). While we agree that current injection is a valid and simpler way to approximate the effect of a bAP, we opted for a purely dynamical approach. Our method offers a more nuanced view of the interaction between the somatic and dendritic compartments. In addition, we are currently attempting to construct a more biologically plausible learning mechanism that does not relay on the hand-waving transient boosting of the somato-dendritic coupling. Therefore, we do not pursue the possibility suggested by the reviewer in the revised manuscript.

3. My final major comment pertains to the spike-rate after learning. It appears that once a pattern has been learned, the firing rate is extremely high during the presentation of the pattern, with the neuron(s) spiking nearly continuously during the presentation. This does not appear physiological. Is there a way to tune the model, such that a more physiological firing rate is reached after pattern learning?

We thank the reviewer for his careful reading. Indeed, we actively searched the mechanism that enables neurons to continuously fire during the presentation of patterns but not to respond briefly to a certain portion (typically, the onset of a pattern). We asked the neuron to report the entire duration of each pattern as well. For instance, the absence of NMDAR enabled us to model such a brief response. We may include spike-frequency adaptation mechanism to lower the firing rate during pattern presentation. However, we feel it is a secondary problem and did not explore such a mechanism.

Minor comments:
On page 2: "At least, two different computational approaches can be made to pattern detection in neurons. In one approach, synaptic learning rules derived from experimental evidence such as spike-timing- dependent plasticity (STDP) are examined for the purpose." These sentence could benefit from an improved formulation (e.g. "can be made" -> "exist in the literature", "are examined" -> "are investigated".

We have revised the manuscript with the reviewer's suggestion. This is on the last paragraph of page 2, in the revised manuscript.

2. On page 2: "The latter tends to perform well on various tasks as the underlying learning rules are designed to optimize their performance in learning" This reads like a circular reasoning.

We appreciate the reviewer's sharp and fair observation. We have revised the manuscript to improve clarity on how the learning rules lead to good performance. This is on the last paragraph on page 2, in the revised manuscript.

3. On page 3: "well-based". Do the authors mean "well defined"?

We appreciate the reviewer's suggestion. Our intention was to motivate that these particular learning rules are based on solid principles ("well-founded"). According to the reviewer's

comment, we have revised the expression in the manuscript. This is on paragraph 2 of page 3 in the revised manuscript.

4. On page 5: "…for these and similar approaches, including ours…" -> formulation could be simplified.

We have simplified the expression in the revised manuscript on the section "Spike trace-based model for self-supervised pattern detection" on page 5 in the revised manuscript.

5. On page 5: "… 2) postsynaptic potentials (PSPs) evoked by presynaptic spikes (Fig. 1B),…" figure 1B doesn't show PSPs.

We appreciate the reviewer's observation. We have revised the manuscript to formally separate the PSPs (a learning rule component) from presynaptic spikes (what's visualized) on the last paragraph of page 5 in the revised manuscript.
