# Supplementary material for: Transient boosting of action potential backpropagation for few-shot temporal pattern learning
Source: PLoS Comput Biol. 2025 Dec 5;21(12):e1013777. doi: 10.1371/journal.pcbi.1013777 (PMC12698000; doi:10.1371/journal.pcbi.1013777)
Supplement: Attachment — Submitted filename: Response to Reviewers.pdf [file pcbi.1013777.s012.pdf]

Point-to-point reply to the reviwer's comments

Comments by Reviewer #2:
I thank the authors for providing their replies to my concerns. However, at the moment, I do not feel that they have been addressed adequately. Pertaining concerns (1-2), I still find the dynamic coupling mechanism to be poorly motivated. The cited references (62, 91) certainly pertain to the backpropagation of APs, but do not appear to suggest a dynamic somato-dendritic coupling conductance. I insist on this issue, because I think it may be crucial for the functioning of the model, and also related to my 3rd major concern about the high firing rates. In the parameter table, the spike threshold voltage is stated to be -50 mV. As the model is an I&F one, this means that the somatic compartment can not drive the dendritic compartment to a higher voltage; even at infinite coupling, the soma could only drive the dendrite to -50 mV at most. Conversely, this means a high number of spikes is required to elicit a sufficient dendritic calcium response. If the bAP would be modelled in a different way, e.g. through a spike-triggered dendritic current, the dendritic bAP amplitude could be tuned independently of the somatic threshold, leading to a calcium response that could be achieved for lower firing rates. As it stands, it appears that this strong firing rate increase is necessary to achieve a sufficient calcium response and eligibility signal (cf. Fig 2). It should also be noted that in a more physiologically plausible situation, potassium currents would shut off the calcium response as well as the high firing rates. Together, this makes me question whether the described few shot learning hinges crucially on this high firing rate, and whether it could be achieved under more plausible response characteristics. In their reply, the authors refer to using adaptation and a more plausible learning mechanism in future work to achieve the few-shot learning with more physiological response characteristics. My issues with this is that I can not assess this claim. I therefore maintain my recommendation of major revision, and feel the authors should either point out the flaw in my reasoning or provide a better exploration of these different settings. This would also make the paper more generalisable.

Response to Reviewer #2:
We thank the reviewer for the detailed comments and for highlighting potential concerns regarding the somato-dendritic coupling. Indeed, the comments were useful for improving the clarity of our manuscript.

In our calcium-based model, the somatic compartment does contribute to dendritic depolarization during the brief post-spike interval. However, we noticed that the previous description of the calcium-based model did not provide sufficient details about its discharging process. Therefore, we have revised the section "*Somatic compartment (calcium-based)*" (p39 of the revised manuscript) in which we have explicitly described how V_s drives V_d after V_s reaches to firing threshold and have addressed the concerns regarding the somato-dendritic coupling and its role in calcium-dependent plasticity. Below, we explain these points.

When the soma reaches to threshold (-50mV), the somatic voltage V_s is instantaneously elevated at V_{peak} (+30mV) representing the peak spike amplitude in our model. Then, the soma enters a refractory period during which synaptic and dendritic input is no longer integrated and the somatic potential is rapidly hyperpolarized (Eq. 21, with the increased leak g_{lsr}). However, since the somatic voltage V_s remains sufficiently high for a certain duration after spike firing, it produces a positive driving force $V_s - V_d$ on the dendritic compartment through the coupling conductance g_{csd}. This wave-like current I_{csd} transiently depolarizes the dendrite, recruits calcium influx, and induces plasticity in synapses active at this spike event. Therefore, the dendritic depolarization does **not** require continuous high firing to elicit a sufficient dendritic calcium response: it is driven by the post-spike somatic voltage in a spike-time-locked manner. The following figure further demonstrates the time courses of V_s and V_d explained above for the calcium-based model.

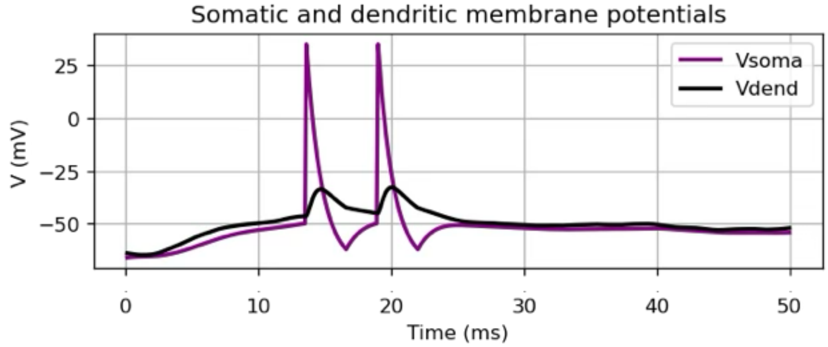

The reviewer's suggestion of a spike-triggered dendritic current independent of the somatic voltage is indeed an alternative approach. However, in this study, we adopted a formulation that minimally extends the Integrate and Fire framework.

In the section "*Somatic compartment (calcium-based)"* of the revised manuscript, we have mentioned that the current parameterization requires more than one spike to elicit sufficient dendritic calcium, and that additional biophysical mechanisms (e.g., potassium currents, adaptation) could reduce this requirement. However, please note that continuous high firing is a consequence of synaptic plasticity but not the opposite, and indicates the entire period of a temporal community in presynaptic inputs. We hope the revised manuscript clarifies the concerns raised by the reviewer.
