# Supplementary material for: Transient boosting of action potential backpropagation for few-shot temporal pattern learning
Source: PLoS Comput Biol. 2025 Dec 5;21(12):e1013777. doi: 10.1371/journal.pcbi.1013777 (PMC12698000; doi:10.1371/journal.pcbi.1013777)
Supplement: Attachment — Submitted filename: Review on PCOMPBIOL-D-24-01766.docx [file pcbi.1013777.s009.docx]

**Review on**:

*Transient Boosting of Action Potential Backpropagation for Few-shot Temporal Pattern Learning*

**Summary**:

The paper introduces a self-supervised plasticity rule for few-shot pattern learning. The plasticity rule consists of three parts in a two-compartment neuron model: the self-supervised error signal resembles the sliding window of BCM-rule except with eligibility trace of spike trains; the post-synaptic potential is eligibility trace of total input; the scaling term is a boundary factor for synaptic weight.

The original form is extended to a calcium-based form where the concentration of calcium serves as the substrate for eligibility traces and the error signal is mediated by bAP, i.e., somato-dendritic coupling. The calcium-based form is biologically plausible, and the simulations fit with single-doublet pairing protocol induced Schaffer-collateral STDP in hippocampus. The performance of pattern learning is further improved by the insertion of NMDARs which promotes bursting activities.

Lastly, the paper proposes a recurrent scheme with preconfigured small assemblies. The original form efficiently learns the input pattern with just several shots by recruiting the pre-existing assemblies.

The paper in general did solid modeling and simulations. The idea of using calcium as the substrate for eligibility trace is interesting. However, there are some subtle assumptions that need further checking, especially the motivation for the complexity of models. Next page are the major comments regarding these assumptions and model choice.

In the last page, I have some minor comment line by line.

**Major comments**:

1. In all settings, the feedforward(input) connections are all-to-all. This would nevertheless induce some level of initial bias in network response, e.g., some neurons would have higher response to certain input patterns. How strong is this initial bias in your model? In addition, how sensitive is performance to initialization of weights? (e.g., instead of gaussian, log-normal or uniform)
2. The homeostasis has extremely fast updating (time constant of 1.0ms), this does not align with experiments that homeostasis is usually of a much longer time scale.
3. In eq.18, an extra low-pass filter (time constant 100ms) is used for PI(i,t). Is this necessary as PI(i,t) itself is the product of filtered signals e(t) and PSP(i,t)?
4. In the Ca-NMDARs model, the AMPA/NMDA ratio is chosen as 5%. Is there experimental support or just to have minimal effect on dend depolarization? In addition, the effect of NMDARs is to promote bursting. What is the difference between NMDARs insertion and explicitly setting 10% input neurons to burst?
5. There is no mentioning of synaptic delay in either the single neuron or network model. How is it defined? Would synaptic delay change your results?
6. In the recurrent network model, the connectivity is set to a scheme where inhibition is strongly recruited by excitatory neurons (Exc-Inh is very strong). It looks like a trivial choice. Is this a necessary condition?
7. The authors argue that the pre-existing assemblies provide initially active neurons. What about the pre-existing within-assembly connectivity? Would random connectivity with some neurons being intrinsically more active give similar results?
8. A general concern is there are so many parameters hence different combinations could lead to the same results(redundancy). How do you argue for the choice of conductance and time constants? More, specifically how do you justify the relative value of different conductance and time constants?

**Detailed comments:**

1) Page 6 Eq. 17, the Alpha function gives rise to typical spine head volume distribution under what condition?
2) Page 6 bottom lines, why equal number of exc and inh input neurons?
3) Page 7 paragraph 2 last sentence, what is the distribution of early and late onset responses?
4) Page 8 paragraph 1, how is the long-tailed strength distribution related to bimodality of trained weights?
5) Fig1.F, e(t) is always on(non-zero). What happens if there is a period of silence? Would it drastically change weights?
6) Page 10 paragraph 2 last line, LTP and LTD become equivalent. Is it strictly or roughly equivalent?
7) Page 13 paragraph 2, what is opposite conductance of the somato-dendritic coupling?
8) Page 13 second-last line, is it “(Supp Fig. 3B and 3D)”?
9) Fig.2D, add units to color bar.
10) Fig.3B, the confidence interval of mean estimation? In Fig2.C, the variance seems very large, therefore is the mean estimation meaningful?
11) Page 18 paragraph 2, what is the use of synaptic transmission failure? Simply adding more noise to the Poisson spike trains?
12) Page 28 paragraph 1 last line, with or without?
13) Page 28 paragraph 2, last line, be hinted at?
14) Fig.6B, the raster plot is a bit messy. What does pair-wise correlation look like?
15) Fig.6E, does the normalized spike count increase because of decrease of assembly size?
16) Page 3 paragraph 2, (but see) what?
17) Eq 31, is it Ica (+=) or a different variable Ica+ from Eq 26?
